# 

**Published:** 1851-10

**Authors:** 


### CONTENTS OF THE DENTAL REGISTER OF THE WEST.

### OCTOBER, 1851.

### PART I.—ORIGINAL COMMUNICATIONS.

PAGE.
Dr. Griffith's Address, ........................ 1
Proceedings of the Miss. Valley Association of Dental Surgeons, 6
Practical Thoughts on Tooth Drawing, by C. T. Cushman, D. D. S., 26

### PART II.—SELECTIONS.

Treatment of Dental Caries, etc., by Robert Authur, D. D. S., .... 34
"World's Fair," from Dental News Letter, .................... 51

### PART III.—EDITORIAL.

A Trip on the Railroad, .................................... 57
New York World's Fair, .................................... 59
Dental Times and Advertiser, .............................. 59
The Mississippi Valley Association of Dental Surgeons, ......... 59
American Society of Dental Surgeons, ........................ 60
Society Dental Surgeons State of New York, .................. 60
Enlargement of the "Register," .............................. 60
To our Subscribers, ......................................... 60
Dental Colleges, ............................................ 60
Treatment of Dental Caries, etc., ........................... 61
Extraction of Teeth, by Dr. Cushman, ........................ 62
Specimens for Museum of Ohio College of Dental Surgeons, .... 62

